# Evaluating the mutagenicity of *N*-nitrosodimethylamine in 2D and 3D HepaRG cell cultures using error-corrected next generation sequencing

**DOI:** 10.1007/s00204-024-03731-4

**Published:** 2024-04-07

**Authors:** Ji-Eun Seo, Yuan Le, Javier Revollo, Jaime Miranda-Colon, Hannah Xu, Page McKinzie, Nan Mei, Tao Chen, Robert H. Heflich, Tong Zhou, Timothy Robison, Jessica A. Bonzo, Xiaoqing Guo

**Affiliations:** 1 Division of Genetic and Molecular Toxicology, National Center for Toxicological Research, U.S. Food and Drug Administration, Jefferson, AR 72079 USA; 2 Center for Veterinary Medicine, U.S. Food and Drug Administration, Rockville, MD 20855 USA; 3 Center for Drug Evaluation and Research, U.S. Food and Drug Administration, Silver Spring, MD 20993 USA

**Keywords:** NDMA, HepaRG cells, 3D spheroids, Genotoxicity, Mutation, Error-corrected next generation sequencing

## Abstract

Human liver-derived metabolically competent HepaRG cells have been successfully employed in both two-dimensional (2D) and 3D spheroid formats for performing the comet assay and micronucleus (MN) assay. In the present study, we have investigated expanding the genotoxicity endpoints evaluated in HepaRG cells by detecting mutagenesis using two error-corrected next generation sequencing (ecNGS) technologies, Duplex Sequencing (DS) and High-Fidelity (HiFi) Sequencing. Both HepaRG 2D cells and 3D spheroids were exposed for 72 h to *N*-nitrosodimethylamine (NDMA), followed by an additional incubation for the fixation of induced mutations. NDMA-induced DNA damage, chromosomal damage, and mutagenesis were determined using the comet assay, MN assay, and ecNGS, respectively. The 72-h treatment with NDMA resulted in concentration-dependent increases in cytotoxicity, DNA damage, MN formation, and mutation frequency in both 2D and 3D cultures, with greater responses observed in the 3D spheroids compared to 2D cells. The mutational spectrum analysis showed that NDMA induced predominantly A:T → G:C transitions, along with a lower frequency of G:C → A:T transitions, and exhibited a different trinucleotide signature relative to the negative control. These results demonstrate that the HepaRG 2D cells and 3D spheroid models can be used for mutagenesis assessment using both DS and HiFi Sequencing, with the caveat that severe cytotoxic concentrations should be avoided when conducting DS. With further validation, the HepaRG 2D/3D system may become a powerful human-based metabolically competent platform for genotoxicity testing.

## Introduction

Mutagenicity testing is an important element of product safety evaluations due to the close relationship between mutation and cancer, as well as other genetic disorders (Paul et al. 2019). Currently, there are five OECD TGs (the Organization for Economic Co-operation and Development Test Guidelines) available for assessing the mutagenic potential of environmental exposures using different in vitro and in vivo mutagenicity assays (OECD 2016a). Among in vitro assays, the bacterial reverse mutation test (the Ames test, OECD TG471) detects point mutations including base pair substitution and frameshifts using *Salmonella typhimurium* and *Escherichia coli* bacterial tester strains. The mammalian cell gene mutation tests (OECD TG476 and 490) detect forward mutations in reporter genes; specifically, the endogenous *hypoxanthine–guanine phosphoribosyl transferase* gene (*HPRT*), the *xanthine-guanine phosphoribosyl transferase* transgene (*XPRT*), and the *thymidine kinase* gene (*TK*). In vivo gene mutation assays include transgenic rodent (TGR) somatic and germ cell gene mutation assays (OECD TG488) and the recently developed erythrocyte *Pig-a* gene mutation assay (OECD TG470). Both assays are conducted in rodents and generally employ repeated dose administrations of the test substance.

Mutation tests detect different types of genetic changes, i.e., base pair substitutions, frameshifts, indels, and chromosomal events, depending on the assay (Mei et al. 2014; OECD 2015). The most commonly used mammalian cells for the in vitro tests include human lymphoblastoid TK6 cells, mouse lymphoma L5178Y cells, Chinese hamster ovary (CHO) cells and lung V79 cells (Li et al. 2020; Verheyen et al. 2017). These cell lines grow reliably and relatively rapidly in culture, but they are all deficient in the metabolic activation of test substances. For mutagens that require metabolic activation, an exogenous activation system (e.g., liver S9 prepared from rats pretreated with enzyme inducers) is usually employed. Thus, species differences in metabolism and the artificial nature of the S9 system are a concern (Obach and Dobo 2008; Robison and Jacobs 2009).

Human-derived cells that contain defined Phase I and Phase II metabolic enzymes may provide more-relevant metabolic activation and improve the predictive value of in vitro assays for evaluating genotoxicity in humans (Guo et al. 2020a; Kirkland et al. 2007). The human hepatoma HepaRG cell line expresses metabolic enzymes at levels comparable to those found in primary human hepatocytes (PHHs) and is considered a promising cell line for evaluating human in vivo toxicity (Donato et al. 2022; Tascher et al. 2019). Differentiated HepaRG cells have limited proliferative capacity but can be used for detecting DNA damage using the comet assay (Josse et al. 2008). Additionally, the cells can be adapted for the micronucleus (MN) assay by stimulating them to replicate by supplementing the culture medium with human epidermal growth factor (hEGF) (Josse et al. 2012). Currently, the HepaRG 2D/3D systems have been used with the comet and MN assays to assess the genetic toxicity of a variety of compounds, including carcinogens, pyrrolizidine alkaloids, nanomaterials, and food contact recycled paperboard extracts (Allemang et al. 2018; Barranger and Le Hegarat 2022; Buick et al. 2020; Jalili et al. 2022; Mandon et al. 2019; Souton et al. 2018). HepaRG cells are superior to HepG2 cells for detecting genotoxicants or carcinogens in the comet and MN assays, with three-dimensional (3D) HepaRG spheroids being more sensitive in detecting genotoxicants that require metabolic activation than cells cultured in the 2D format, likely due to the higher levels of cytochrome P450 (CYP) gene expression and enzyme activities in the spheroids compared to its 2D counterpart (Guo et al. 2020b; Seo et al. 2022, 2023a). Building upon our success performing the MN assay in both 2D and 3D HepaRG cultures (Seo et al. 2023a), we extended our work to measure mutation induction in 2D and 3D HepaRG cell models after chemical exposure.

Error-corrected next-generation sequencing (ecNGS) has reduced NGS error rates from 10^‒2^–10^‒3^ to 10^‒7^ or lower (Salk et al. 2018), making it possible to identify and quantify rare mutations on the basis of sequence changes in DNA (Marchetti et al. 2023). ecNGS has potential advantages over classic mutagenesis assays that rely upon reporter genes and genetically modified animals, and been proposed as a promising alternative approach in replacing the traditional mutagenicity assays recommended by the OECD (Dodge et al. 2023). Currently, several ecNGS technologies are available, e.g., the Safe Sequencing System (SafeSeqS) that uses single-stranded molecular barcodes in the tails of PCR primers, Duplex Sequencing (DS) that ligates molecular barcodes to both strands of double-stranded molecules, and PacBio High-Fidelity (HiFi) Sequencing that uses long-read sequencing platforms to repeatedly determine the sequence of both DNA strands of circularized molecules (Revollo et al. 2021; Salk and Kennedy 2020). DS targets an optimized set of twenty 2.4-kb representative genomic regions (a total target size of 48 kb) spread across the autosomes of the human genome and sequences at relatively high depth; whereas HiFi Sequencing examines the majority of the human genome for mutation and sequences at relatively low depth (Cho et al. 2023; Miranda et al. 2022). Both DS and HiFi Sequencing have been successfully employed in our previous studies with other in vitro cell models (Revollo et al. 2021; Wang et al. 2021). In this proof-of-principle study, we treated both 2D and 3D differentiated HepaRG cell models with *N*-nitrosodimethylamine (NDMA), a prototypical mutagen, and measured mutation using the two high accuracy ecNGS technologies, DS and HiFi Sequencing.

NDMA was chosen for the present study because it is a well characterized mutagen that requires metabolic activation to exert its genotoxic and carcinogenic effects (IARC 1978; Kirkland et al. 2016). In addition, there currently is renewed interest in the genotoxicity of NDMA due to its recent identification as an impurity in several popular human drugs used for the treatment of common diseases such as hypertension, heartburn, and diabetes (FDA 2021; Seo et al. 2023b). Our previous studies confirmed that NDMA induced significant DNA strand breakage and MN formation following a 24-h treatment of both 2D and 3D HepaRG cell models without the addition of liver S9 fractions (Seo et al. 2022, 2023a, 2023b). The present study assessed NDMA-induced mutations following a 72-h treatment in both 2D and 3D HepaRG models with further optimized DS and HiFi Sequencing techniques.

## Materials and methods

### 2D and 3D HepaRG models

**Fig. 1:**
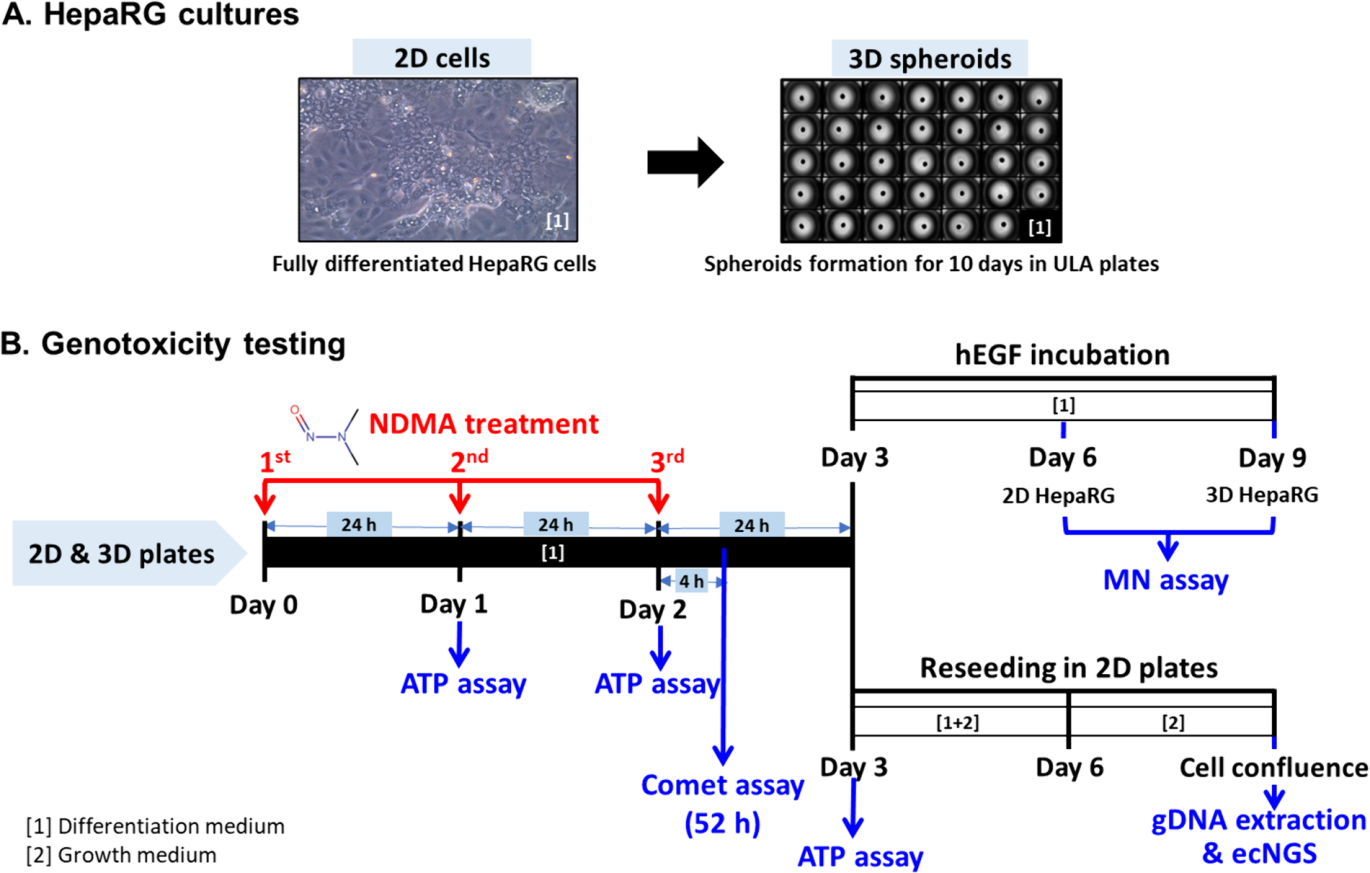
2D and 3D HepaRG cultures and workflow for genetic toxicity testing. **A** HepaRG cells were cultured in growth medium for 14 days (proliferation status) and then in differentiation medium for another 14 days (fully differentiated status). Differentiated cells were plated into a 384-well ultra-low attachment (ULA) plate at a density of 3 × 10^3^ cells/well to form 3D spheroids. **B** 2D cells at Day 3 and 3D spheroids at Day 10 were exposed to various concentrations of NDMA for 72 h. The comet assay was conducted at 4 h after the 3^rd^ treatment medium was refreshed (i.e., 52-h treatment). The MN assay was conducted following an additional human epidermal growth factor (hEGF) stimulation period (i.e., 3 days for 2D or 6 days for 3D) after the 72-h treatment. For the error-corrected next-generation sequencing, both 2D and 3D cultures were reseeded and grown in culture medium until confluence (90%). [1], differentiation medium; [2], growth medium

Both 2D and 3D HepaRG models were generated as previously described (Seo et al. 2022). Briefly, human hepatoma HepaRG cells (Cat# HPR101, Biopredic International, Saint Grégoire, France) were cultured for a total of 28 days according to the supplier’s protocol, i.e., first in growth medium for 14 days followed by 14 days of differentiation in differentiation medium, both incubations conducted at 37 °C in a humidified atmosphere of 5% CO_2_ in air. The growth medium was prepared by adding growth additives (Cat# ADD711C, Lonza, Walkersville, MD) to William’s E Medium (Cat# 12551032, Thermo Fisher, Waltham, MA) supplemented with 2 mM GlutaMax (Cat# 35050061, Thermo Fisher) and 100 μg/ml primocin (Cat# NC9141851, InvivoGen, San Diego, CA). The differentiation medium was prepared the same way as the growth medium except for replacing the growth additive with differentiation additive (Cat# ADD721C, Lonza). For initiating 2D cultures, fully differentiated HepaRG cells were dissociated with TrypLE Express (Cat# 12604039, Thermo Fisher) and reseeded into each well of 6-well and 96-well flat bottom plates at densities of 1.5 × 10^6^ and 5 × 10^4^ cells per well, respectively. For preparing 3D spheroid cultures, the dissociated cells were plated into each well of 384-well ultra-low attachment (ULA) round-bottom plates (Corning Inc., Corning, NY) at a density of 3 × 10^3^ cells/well (Fig. 1A). The plates were maintained at 37 °C in a humidified atmosphere with 5% CO_2_ and the medium was refreshed every 2–3 days. An automatic pipetting system, the VIAFLO 96/384 Electronic Pipette (Integra Biosciences, Hudson, NH), was used to change media in the ULA plates to avoid spheroid disturbance.

### Exposure of HepaRG cells to NDMA

Stock solutions were prepared freshly before each treatment by diluting NDMA (Cat# PHR2407, Sigma-Aldrich, St. Louis, MO) in deionized water. Working concentrations (100 ×) then were prepared by serial dilution in differentiation medium. Three days or 10 days after initiating the cultures, 2D HepaRG cells or 3D spheroids were exposed to NDMA for 72 h, with the treatment medium changed three times at 0, 24, and 48 h (Fig. 1B). For the first treatment in ULA plates on Day 0, half of the medium was carefully removed from each well and then a half volume of 2 × final concentrations of NDMA were added to the spheroid cultures. The treatment medium in ULA plates was replaced with half of 1 × the final concentrations of NDMA for the second and third treatments on Day 1 and Day 2, respectively. The final concentrations of NDMA were 1, 5, and 10 mM for 2D cultures and 0.1, 0.5, 1, and 2 mM for 3D spheroids. The concentrations were informed by previous studies (Seo et al. 2022, 2023a) and were chosen to allow observation of a wide range of biological responses.

### Cytotoxicity assay

Cytotoxicity was evaluated with the CellTiter-Glo ATP-based assay on Day 1, Day 2, and Day 3 following 24-, 48-, and 72-h of NDMA treatments, respectively (Fig. 1B). The reagent in the CellTiter-Glo Luminescent Cell Viability Assay kit (Cat# G7572, Promega; Madison, WI) for 2D cultures or the CellTiter-Glo 3D Cell Viability Assay kit (Cat# G9683, Promega) for 3D spheroids was added to wells of the 96-well plate at a ratio of 1:10. One spheroid per well was used for the 3D ATP assay. Following a 10-min incubation at room temperature, luminescence was measured with a Cytation 5 Cell Imaging Multi-Mode Reader (BioTek, Winooski, VT). The relative cell viability was expressed as the percentage of luminescent signal produced by the treated cells as compared to the untreated control cells.

### Alkaline comet assay

Four hours after applying the 3rd NDMA treatment (i.e., after 52-h of exposure), DNA damage was evaluated by the comet assay following the manufacturer’s protocol (Trevigen, Gaithersburg, MD). Briefly, 2D HepaRG cells and 3D spheroids were dissociated with TrypLE Express into single cell suspensions. After washing with ice-cold phosphate-buffered saline (PBS), the cells were mixed with 1% low melting point agarose (Cat# BP165-25, Thermo Fisher) in PBS (> 1 × 10^5^ cells/ml) at 37 °C and spread evenly across wells of comet slides (Cat# 4250–200-03, Trevigen) (Ali et al. 2014). The slides were placed flat at 4 °C for 10 min to solidify the agarose and then immersed in pre-chilled lysis solution (Cat# 4250–050-01, Trevigen) overnight at 4 °C in the dark. The next day the slides were transferred to freshly prepared alkaline solution (pH > 13) for 1 h at 4 °C to denature and unwind the DNA. Electrophoresis was performed at 21 V for 30 min in the same alkaline solution at 4 °C in the dark. The slides were rinsed with distilled water, fixed in 70% ethanol, and stained with SYBR Gold (Cat# S11494, Invitrogen, Carlsbad, CA). Comet images were acquired using a Leica DMI4000 B fluorescence microscope (Leica Microsystems, Buffalo Grove, IL) and the percentage of tail DNA (% tail DNA) was determined using Trevigen Comet Analysis Software (Cat# 4260-000-CS, 4/27/16 rev4).

### Micronucleus (MN) assay

Following the 72-h of treatment, eight spheroids were pooled from the 384-well plates and transferred into each well of a 96-well round-bottom plate (TPP, Switzerland) in quintuplicate. The treatment media for both the 2D and 3D cultures were replaced with 200 μl of fresh differentiation medium supplemented with 100 ng/ml hEGF (Cat# E5036, Sigma-Aldrich) to stimulate cell proliferation. The 2D and 3D 96-well plates were incubated for additional 3 days and 6 days with hEGF, respectively, in order to stimulate 1.5 to 2 cell population doublings (Guo et al. 2020b; Seo et al. 2023a). MN frequency was analyzed using the In Vitro MicroFlow Kit (Cat# In Vitro-1,000/200, Litron Laboratories, Rochester, NY) following the manufacturer’s protocol. The cells were sequentially stained with ethidium monoazide and SYTOX Green to label apoptotic/necrotic cells and chromatin, respectively. Cells were lysed and the MN events were recorded using a BD FACSCanto II flow cytometer (BD Biosciences, San Jose, CA). Results from a total of 10,000 intact nuclei were recorded. The percentage of MN (%MN) was calculated by dividing the MN events by the total number of nucleated events. Precent relative survival (%RS) was used as an indicator of cytotoxicity and calculated as the ratio of nucleated events in treated cells relative to those of untreated controls at a specified time point.

### Genomic DNA extraction

Following treatment of the 2D cultures, three replicate pools of 6 × 10^5^ HepaRG cells were prepared for each treatment concentration and used for genomic DNA (gDNA) extraction. For the 3D cultures, approximately 200 spheroids (for the untreated control, 0.5 and 1 mM NDMA) or 100 spheroids (for 0.1 and 2 mM NDMA) were pooled and dissociated into single cells with TrypLE Express. These cell pools were reseeded in 6-well plates in a 1:1 mixture of growth and differentiation medium and cultured for 3 days (Fig. 1B). Then the medium was changed to growth medium and refreshed every 2–3 days. When confluence was reached in the 6-well plates, the cells were dissociated and replated into 100-mm dishes and cultured until 90% of surface was covered by the cell monolayer. Based on cell counting conducted at this stage, HepaRG cells from both the 2D and 3D cultures had gone through approximately 3–4 cell divisions before gDNA was extracted using a DNeasy Blood & Tissue Kit (Cat# 69,506, QIAGEN, Valencia, CA) with RNase A treatment. The DNA concentration was measured using a Qubit dsDNA Broad Range Assay Kit (Cat# Q32850, Thermo Fisher).

### Duplex Sequencing (DS) and data analysis

DS library preparation was performed using the TwinStrand Duplex Sequencing Human Mutagenesis Kit (Cat# 06-1005-02) according to the manufacturer's protocol (Rev 1.1; TwinStrand Biosciences, Seattle, WA). Briefly, 750 ng of gDNA were enzymatically fragmented (~ 200 bp), end‐repaired, A‐tailed, ligated with DuplexSeq Adapters, and conditioned with Library Conditioning Mix. The library samples then were amplified using unique dual indexing primer pairs for PCR, followed by hybrid capture with the Human‐50 Mutagenesis Panel (Rev. 1.0; TwinStrand Biosciences). Following the first hybrid capture, the samples were amplified with P5/P7 primers, and enriched by a second hybrid capture, followed by the final PCR. Final libraries were quantified using a TapeStation (Agilent, Santa Clara, CA), pooled, and sequenced using 150 bp paired-end reads on an Illumina NovaSeq6000 S4 system (Illumina, San Diego, CA), with a target of 500 million raw reads per sample. The passing filter raw reads for each sample ranged from 328,369,514 to 605,720,702 for 2D HepaRG cells and from 319,443,032 to 458,349,752 for 3D spheroids, with a mean on-target duplex depth of ~ 20,000 ×.

Sequencing data (FASTQ files) were analyzed with the TwinStrand DuplexSeq Mutagenesis App (Version 4.1.0) using the human reference genome version GRCh38 (v3.3) as described in the manual. By default, the App counts only single nucleotide variants with variant allele frequency (VAF) values of less than 1%, which provides a minimum mutation frequency (MF_DS_Min_). Additionally, the maximum mutation frequency (MF_DS_Max_) was generated by reanalyzing the .mut files using the Mutagenesis Report App (Version 4.1.0). In contrast to MF_DS_Min_, MF_DS_Max_ counts multiple identical mutations at the same position (most likely clonally expanded mutations) as independent mutation events.

### HiFi Sequencing and data analysis

Libraries, indexing, and sequencing were performed as previously described (Miranda et al. 2022). Briefly, for each sample, 1 μg of gDNA was sheared to ~ 6-kbp fragments using g-tubes (Covaris, Woburn, MA) and purified with PacBio AMPure beads at a 0.47:1 ratio of beads to sample. The sheared and purified DNA (300 ng) was processed as described by the “Procedure & Checklist—Preparing Multiplexed Microbial Libraries Using SMRTbell Express Template Prep Kit 2.0” protocol (101–696–100 v8, November 2021, PacBio, Menlo Park, CA) using indexes from the Barcoded Overhang Adapter kit 8A (Cat# 101–628–400, PacBio). Three indexed libraries representing the same concentration/condition were combined and sequenced in one 8M SMRT Cell with Polymerase 2.2 for 30 h in a Sequel II instrument following the instructions provided by the “HiFi reads” application with adaptive loading (reagents and sequencer were from PacBio). After sequencing, PacBio complementary forward and reverse consensus reads (generated under default settings by SMRT link v10.1) from each sample were aligned by the Burrows-Wheeler Aligner (BWA) to the hg38 human reference genome. Unique variants in the dataset (i.e., from all samples/conditions) were considered mutations when primary alignments (SAM flags 0 or 16) with mapping qualities (MQ) ≥ 60 were present in both complementary forward and reverse consensus reads and did not exhibit allelic fractions ≥ 5% in a processed Illumina dataset of the corresponding parental HepaRG culture. Finally, filters removed reads (and their corresponding mutations) that contained more than 1 mutation. Illumina libraries of the parental HepaRG culture were prepared as suggested by the Nextera DNA Flex Library Prep kit (Cat# 20060060) and paired-end sequenced (76 × 2) with 150-cycle kits on a NextSeq 500 instrument until whole genome coverages ~ 30-fold were attained from the processed Illumina dataset (aligned to hg38 by BWA, sorted by Samtools, and marked for duplicates by Picard) (kits and sequencer were from Illumina, San Diego, CA). De-indexing was performed as previously described (Miranda et al. 2023).

### Statistical analysis

Data are presented as the mean ± standard deviation (SD) from at least triplicate samples. Statistical significance was evaluated by one-way analysis of variance (ANOVA) followed by Dunnett’s test for comparing the genotoxicity data between treatment groups and the untreated control group (SigmaPlot 13.0, Systat Software, San Jose, CA). When the normality test (Shapiro–Wilk) or equal variance test (Brown-Forsythe) failed, the nonparametric Kruskal–Wallis test on ranks was performed. The mutation frequency data were analyzed using a generalized linear model (glm) approach with the quasipoisson distribution. Pairwise comparisons of each treatment group to the control group were performed by the glht algorithm and *p*-values were adjusted by the Bonferroni correction for multiple comparisons. All these steps were carried out in RStudio (Posit Public Benefit Corporation, Boston, MA). Statistical significance was set at *p* < 0.05.

## Results

### NDMA-induced cytotoxicity in 2D and 3D HepaRG cultures

**Fig. 2:**
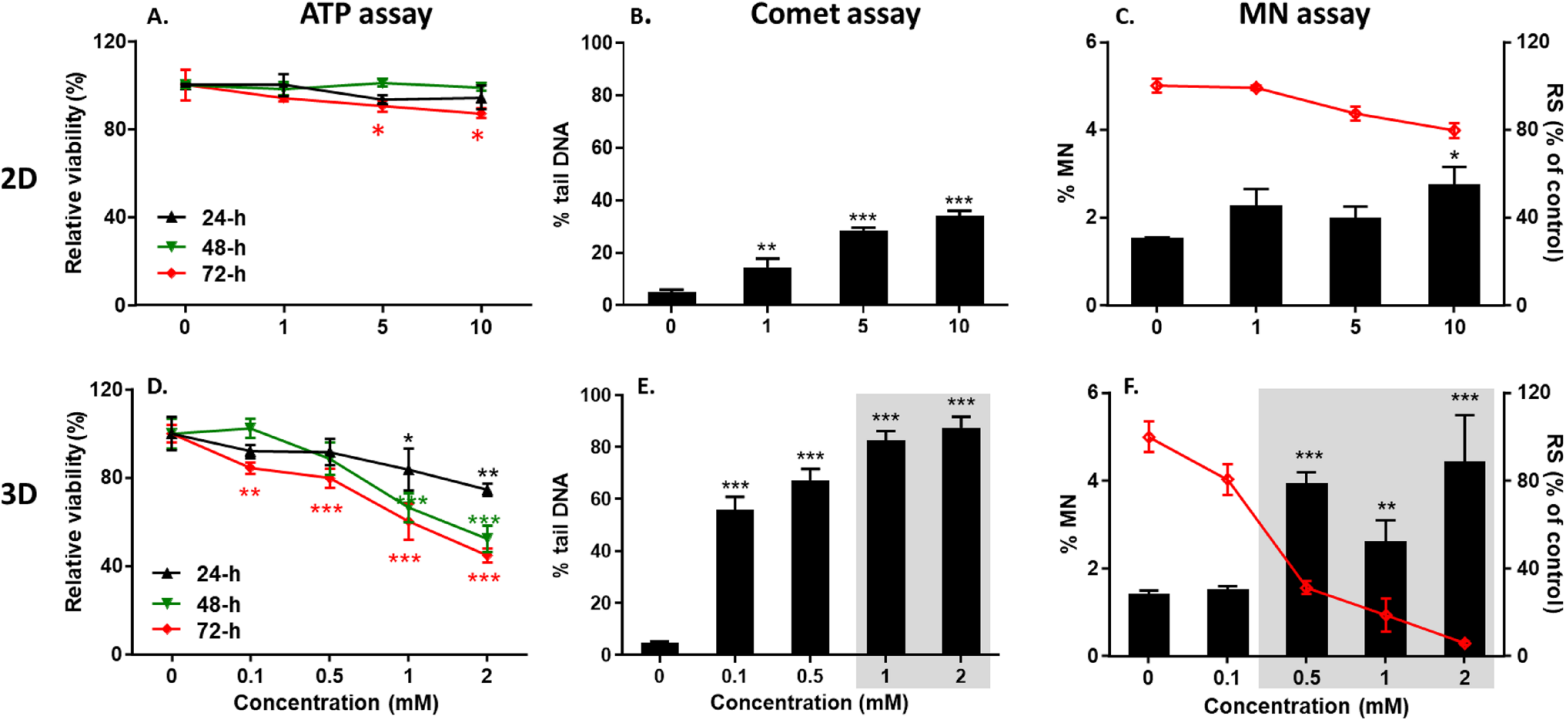
NDMA-induced cytotoxicity and genotoxicity in 2D and 3D HepaRG cultures. HepaRG cells were exposed to NDMA for 72 h. The relative cell viability (% of control) was assessed at 24-h intervals by measuring the ATP levels relative to the vehicle control (**A** and **D**). For the comet assay, 2D HepaRG cells (**B**) and 3D spheroids (**E**) were collected at 4 h after the 3^rd^ treatment medium was refreshed (i.e., 52-h treatment) on Day 2 and DNA damage was expressed as % tail DNA. For the micronucleus (MN) assay, 2D (**C**) and 3D cell cultures (**F**) were exposed to NDMA for 72 h, followed by an additional 3- or 6-day incubation with hEGF (100 ng/ml). MN frequency is presented as the percentage of micronuclei (%MN) relative to intact nuclei [left y-axis and black bar] and cytotoxicity is indicated as the percentage of relative survival (%RS) [right y-axis and red line), as compared to the vehicle control. Gray areas in (**E**) and (**F**) denote NDMA concentrations with levels of cytotoxicity that preclude use of the data for genotoxicity assessment. The data points represented the mean ± standard deviation (SD) (n ≥ 3). Significant difference was determined by one-way ANOVA followed by Dunnett’s test (*p < 0.05, **p < 0.01, and ***p < 0.001 *vs.* vehicle control)

The cytotoxicity of NDMA in 2D and 3D HepaRG models was evaluated daily over the three-day treatment period by the ATP assay. In 2D HepaRG cells, NDMA showed significant cytotoxicity only at the highest two concentrations (5 and 10 mM) following the 72-h treatment. There was no reduction in the ATP levels at any concentrations following 24-h or 48-h of exposure (Fig. 2A). In contrast, NDMA induced significant cytotoxicity in 3D spheroids at a concentration of 1 mM after a 24-h exposure (Fig. 2D). In spheroids, NDMA significantly decreased ATP levels at a concentration as low as 0.1 mM following 72-h of exposure. Compared to the untreated control, the relative cell viability of the spheroids was 84%, 80%, 60%, and 45%, respectively, following 72-h of exposure to 0.1, 0.5, 1, and 2 mM NDMA.

### NDMA-induced DNA damage in 2D and 3D HepaRG cultures

DNA damage induced by NDMA was assessed following a 52-h exposure on Day 2 (Fig. 1B). Both 2D and 3D HepaRG models showed concentration-dependent increases in DNA strand breaks, with NDMA inducing much higher levels of % tail DNA in cells disaggregated from 3D spheroids compared to that of 2D cultures (Fig. 2B and E). Specifically, the treatment with 0, 1, 5, and 10 mM NDMA in 2D cells resulted in 4.9%, 14.5%, 28.5%, and 34.0% tail DNA, respectively. While the treatment with 0, 0.1, 0.5, 1, and 2 mM NDMA in 3D spheroids induced 4.7%, 55.9%, 67.2%, 82.5%, and 87.4% tail DNA, respectively.

### NDMA-induced MN formation in 2D and 3D HepaRG cultures

In 2D HepaRG cells, a 72-h treatment with 10 mM NDMA followed by a 3-day incubation with hEGF resulted in a weak but statistically significant increase in %MN frequency (1.7-fold), with relative survival of 80% (Fig. 2C). The HepaRG spheroids showed significant cytotoxicity at concentrations of 0.5 mM and above after the 72-h exposure followed by the extended 6-day hEGF incubation (Fig. 2F). Nevertheless, 0.5 mM NDMA induced a 3.1-fold increase in %MN frequency in the spheroids. The magnitude of this response may have been augmented by the excessive cytotoxicity in these cultures (OECD (2016b).

### NDMA-induced mutations in 2D and 3D HepaRG cultures

**Fig. 3:**
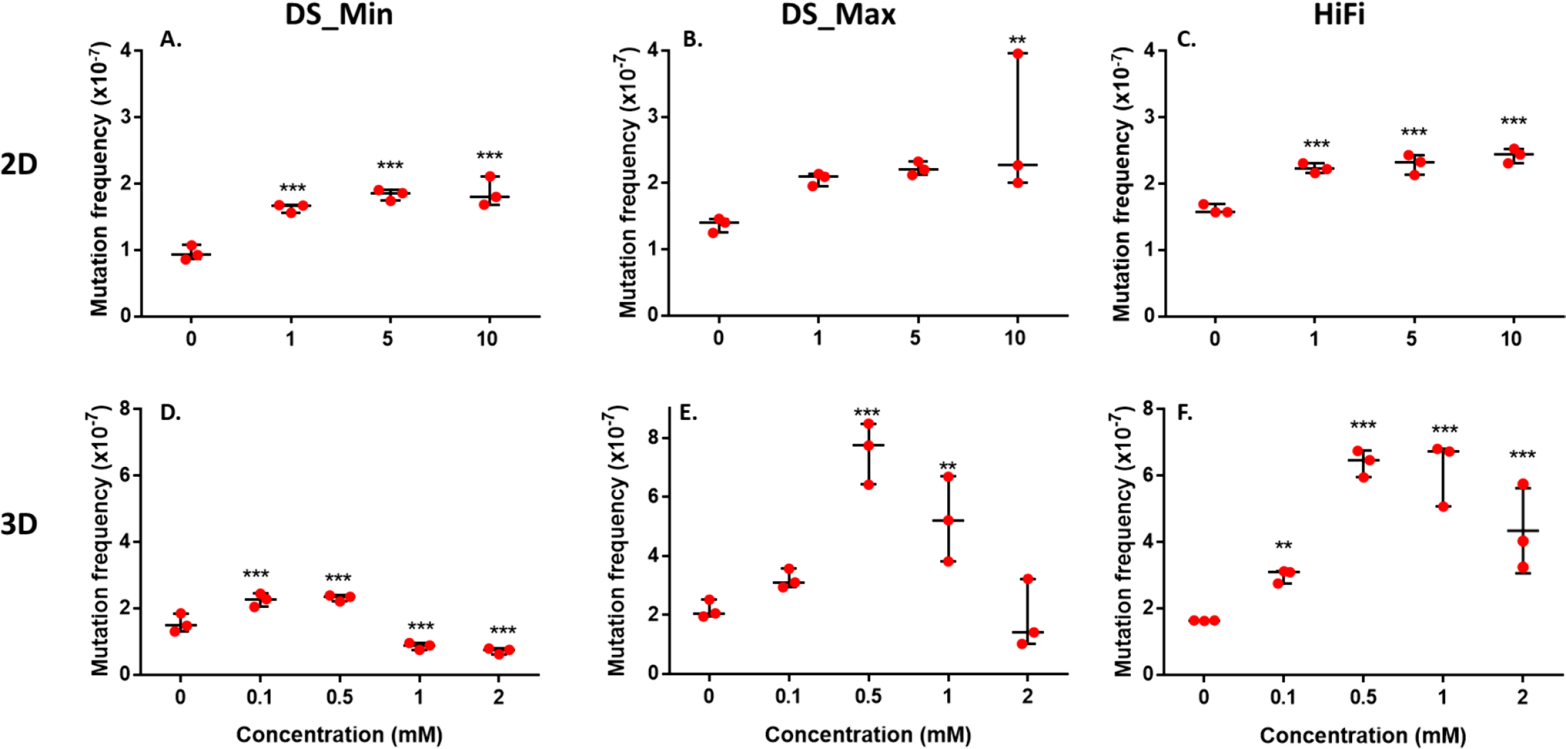
Mutation frequency induced by NDMA in 2D and 3D HepaRG cultures. Mutation was measured in 2D and 3D HepaRG cell models using DS (**A**, **B**, **D**, **E**) and HiFi Sequencing (**C** and **F**). The individual data (n = 3) of mutation frequency (×10^-7^) are expressed as dot plots, along with the mean ± standard deviation (SD). Significant difference was determined by pairwise comparisons between treatment groups and the controls using the glht algorithm with *p *values adjusted by the Bonferroni correction in RStudio (*p < 0.05, **p < 0.01, and ***p < 0.001 *vs*. vehicle control). DS, Duplex Sequencing; Min, the minimum mutation counting method; Max, the maximum mutation counting method

DS performance in the measurement of mutation frequency in 2D and 3D HepaRG cultures are described in Tables 1 and 2, respectively. DS yielded an average of > 1 billion duplex base pairs per sample, with a minimum of 897 million duplex bases sequenced in the spheroids treated with 0.1 mM NDMA (Table 2). The mutation frequency was calculated using both the minimum and maximum mutation counting methods. In 2D HepaRG cells, both methods resulted in a concentration-dependent increase in the frequency of single-nucleotide mutations (Table 1). Statistically significant increases in MF_DS_Min_ were observed in all treated cultures compared with the untreated control (Fig. 3A), and for the highest concentration of 10 mM in MF_DS_Max_ (Fig. 3B). In HepaRG 3D spheroids, when MF_DS_Min_s were compared, 1.5-fold, statistically significant increases in MF were observed in spheroids treated with 0.1 and 0.5 mM NDMA (Table 2A), while the MF_DS_Min_s for 1 and 2 mM NDMA-treated spheroids were significantly reduced (Fig. 3D). When the maximum mutation counting method was used, the MF_DS_Max_s were significantly increased at concentrations of 0.5 and 1 mM NDMA, with the MF induced by 0.5 mM NDMA increasing by 3.5-fold compared to that of the control (Fig. 3E and Table 2B).

**Table 1:** NDMA-induced mutation frequencies in 2D HepaRG cells analyzed by Duplex Sequencing

A. Minimum mutation counting method							
Concentration (mM)	Replicate	Peak tag family size on-target	Duplex bases		MF_min_ (× 10^–7^)	Mean MF_min_ ± SD (× 10^–7^)	Fold change
			Total	Mutations			
0	1	5	1,481,476,441	127	0.86		
	2	17	1,381,279,157	148	1.07	0.95 ± 0.11	–
	3	15	1,168,355,050	108	0.92		
1	1	15	1,234,259,235	207	1.68		
	2	10	1,752,409,763	293	1.67	1.64 ± 0.07*	1.7
	3	10	1,146,296,024	179	1.56		
5	1	12	1,544,212,526	294	1.90		
	2	18	1,426,267,069	265	1.86	1.84 ± 0.08*	1.9
	3	21	1,164,553,481	203	1.74		
10	1	14	1,441,698,879	260	1.80		
	2	15	1,454,228,111	307	2.11	1.87 ± 0.22*	2.0
	3	14	1,317,226,165	222	1.69		

B. Maximum mutation counting method						
Concentration (mM)	Replicate	Duplex Bases		MF_max_ (×10^-7^)	Mean MF_max_ ± SD (×10^-7^)	Fold change
		Total	Mutations			
0	1	1,481,476,441	185	1.25		
	2	1,381,279,157	202	1.46	1.37 ± 0.11	–
	3	1,168,355,050	164	1.40		
1	1	1,234,259,235	264	2.14		
	2	1,752,409,763	367	2.09	2.06 ± 0.10	1.5
	3	1,146,296,024	224	1.95		
5	1	1,544,212,526	359	2.33		
	2	1,426,267,069	314	2.20	2.22 ± 0.10	1.6
	3	1,164,553,481	247	2.12		
10	1	1,441,698,879	327	2.27		
	2	1,454,228,111	575	3.95	2.74 ± 1.06*	2.0
	3	1,317,226,165	264	2.00		

*MF* mutation frequency, *SD* standard deviation

*p < 0.05 *vs*. control

**Table 2:** NDMA-induced mutation frequencies in 3D HepaRG spheroids analyzed by Duplex Sequencing

A. Minimum mutation counting method							
Concentration (mM)	Replicate	Peak tag family size on-target	Duplex bases		MF_min_ (× 10^–7^)	Mean MF_min_ ± SD (× 10^–7^)	Fold change
			Total	Mutations			
0	1	17	1,073,147,757	140	1.30		
	2	18	1,076,718,721	159	1.48	1.54 ± 0.28	–
	3	14	1,060,484,015	196	1.85		
0.1	1	17	896,658,597	204	2.28		
	2	17	916,695,837	224	2.44	2.25 ± 0.20*	1.5
	3	20	974,445,860	199	2.04		
0.5	1	18	951,153,732	210	2.21		
	2	17	1,007,890,013	237	2.35	2.32 ± 0.10*	1.5
	3	19	1,065,498,304	255	2.39		
1	1	13	1,212,024,994	91	0.75		
	2	22	1,021,661,806	98	0.96	0.86 ± 0.11*	0.6
	3	15	1,361,806,550	120	0.88		
2	1	17	1,064,402,479	80	0.75		
	2	16	1,174,527,310	93	0.79	0.72 ± 0.09*	0.5
	3	21	936,163,418	58	0.62		

B. Maximum mutation counting method						
Concentration (mM)	Replicate	Duplex bases		MF_max_ (× 10^–7^)	Mean MF_max_ ± SD (× 10^–7^)	Fold change
		Total	Mutations			
0	1	1,073,147,757	209	1.95		
	2	1,076,718,721	221	2.05	2.17 ± 0.30	–
	3	1,060,484,015	267	2.52		
0.1	1	896,658,597	278	3.10		
	2	916,695,837	327	3.57	3.20 ± 0.33	1.5
	3	974,445,860	286	2.94		
0.5	1	951,153,732	610	6.41		
	2	1,007,890,013	855	8.48	7.55 ± 1.05*	3.5
	3	1,065,498,304	825	7.74		
1	1	1,212,024,994	462	3.81		
	2	1,021,661,806	683	6.69	5.23 ± 1.44*	2.4
	3	1,361,806,550	709	5.21		
2	1	1,064,402,479	343	3.22		
	2	1,174,527,310	165	1.40	1.88 ± 1.18	0.9
	3	936,163,418	95	1.01		

*MF* mutation frequency, *SD* standard deviation

*p < 0.05 *vs*. control

HiFi Sequencing yielded an average of > 4 billion analyzed bases per sample (Table 3). The 72-h treatment with NDMA resulted in concentration-dependent, statistically significant increases in MFs for both the 2D and 3D cultures (Fig. 3C and F). Furthermore, NDMA induced higher MF fold-increases in the spheroids than in the 2D cells (3.9- *vs*. 1.5-fold).

**Table 3:** NDMA-induced mutation frequencies in HepaRG cells analyzed by PacBio High-Fidelity (HiFi) Sequencing

A. 2D HepaRG cells						
Concentration (mM)	Replicate	Bases analyzed		MF (× 10^–7^)	Mean MF ± SD (× 10^–7^)	Fold change
		Total	Mutations			
0	1	4,170,760,000	644	1.54		
	2	3,743,930,000	622	1.66	1.59 ± 0.07	–
	3	4,126,310,000	641	1.55		
1	1	4,646,010,000	1061	2.28		
	2	4,102,640,000	871	2.12	2.19 ± 0.08*	1.4
	3	4,092,280,000	891	2.18		
5	1	3,612,230,000	764	2.12		
	2	4,908,480,000	1140	2.32	2.28 ± 0.15*	1.4
	3	4,712,930,000	1139	2.42		
10	1	4,105,750,000	999	2.43		
	2	3,977,460,000	916	2.30	2.42 ± 0.11*	1.5
	3	3,724,380,000	941	2.53		

B. 3D HepaRG spheroids						
Concentration (mM)	Replicate	Bases analyzed		MF (× 10^–7^)	Mean MF ± SD (× 10^–7^)	Fold change
		Total	Mutations			
0	1	5,348,490,000	864	1.62		
	2	5,108,390,000	832	1.63	1.63 ± 0.02	–
	3	3,944,370,000	649	1.65		
0.1	1	4,442,130,000	1386	3.12		
	2	4,141,730,000	1280	3.09	2.98 ± 0.21*	1.8
	3	3,688,360,000	1010	2.74		
0.5	1	4,676,720,000	2778	5.94		
	2	4,824,020,000	3250	6.74	6.38 ± 0.41*	3.9
	3	4,343,740,000	2810	6.47		
1	1	5,075,170,000	3396	6.69		
	2	5,133,920,000	3426	6.67	6.09 ± 1.03*	3.7
	3	4,316,410,000	2112	4.89		
2	1	4,953,360,000	2589	5.23		
	2	4,834,670,000	1543	3.19	4.04 ± 1.06*	2.5
	3	4,238,120,000	1567	3.70		

*MF* mutation frequency, *SD* standard deviation

*p < 0.05 *vs*. control

### Simple base substitution

**Fig. 4:**
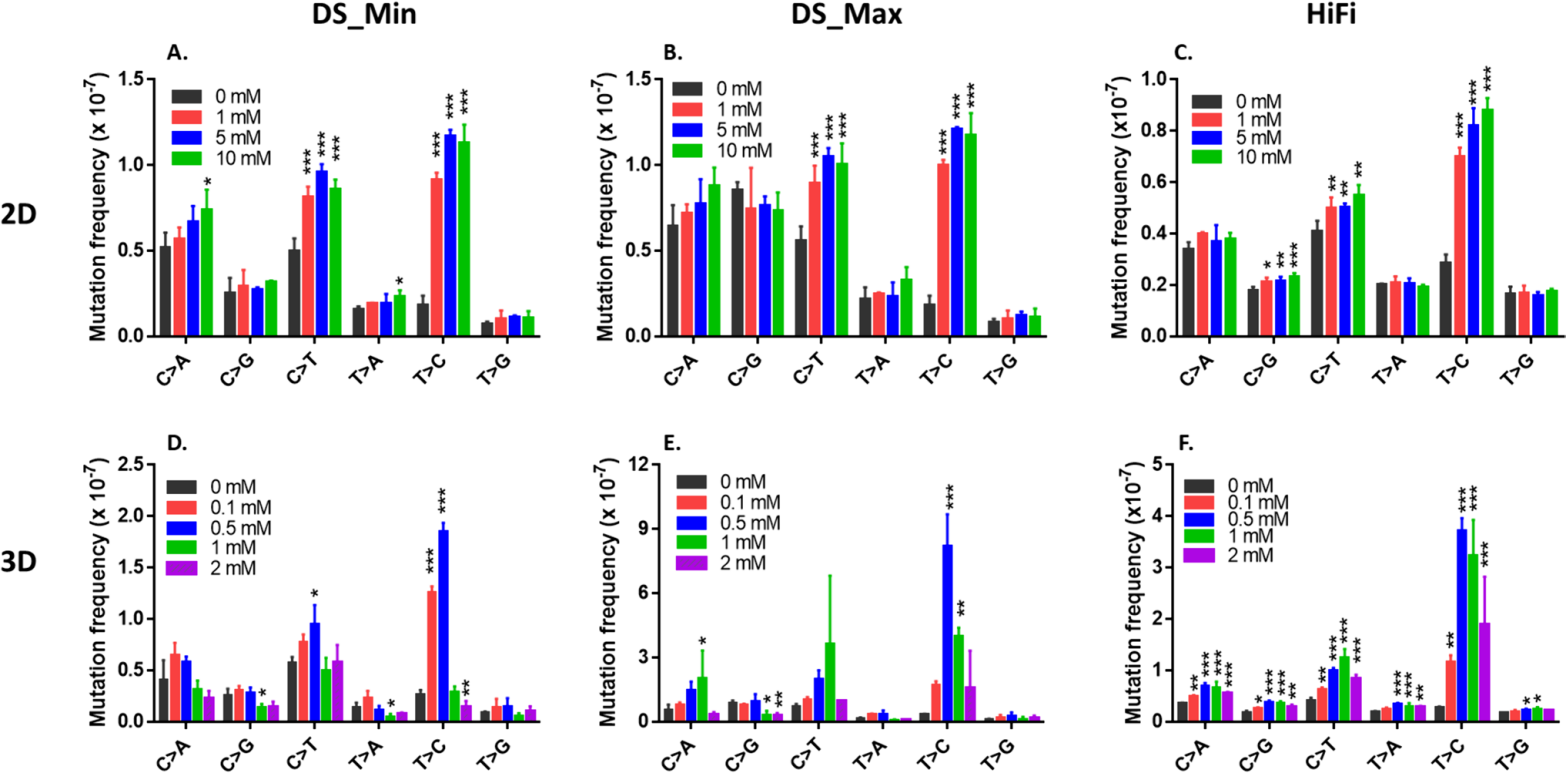
Mutation frequencies of individual base substitution in NDMA-treated 2D and 3D HepaRG cultures. The data are expressed as the mean ± standard deviation (SD) (n = 3). Significant difference was determined by pairwise comparisons between treatment groups and the controls using the glht algorithm with *p* values adjusted by the Bonferroni correction in RStudio (*p < 0.05, **p < 0.01, and ***p < 0.001 *vs*. vehicle control). *DS* duplex sequencing; *Min* the minimum mutation counting method; *Max* the maximum mutation counting method

The simple base substitution spectra provided by DS and HiFi Sequencing indicated that the frequencies of A:T → G:C and G:C → A:T transition increased significantly in cells treated with all concentrations of NDMA in 2D cultures, regardless of the mutation counting method (i.e., minimum or maximum) used in DS (Fig. 4A–C). In addition, HiFi Sequencing detected a significant increase in G:C → C:G transversion at concentrations of 5 and 10 mM in 2D cultures (Fig. 4C).

In 3D spheroids, DS data showed that the frequencies of A:T → G:C transitions increased significantly at the middle concentrations (Fig. 4D and E). In contrast, HiFi Sequencing detected significant increases in all types of base substitutions at almost all concentrations, with A:T → G:C transition being the dominant mutation, followed by G:C → A:T transition (Fig. 4F).

**Fig. 5:**
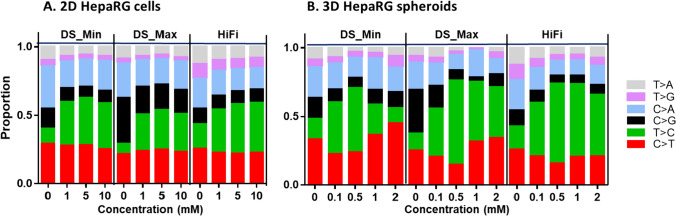
Proportions of individual base substitution in NDMA-treated 2D and 3D HepaRG cultures. The data are expressed as the average proportion of three independent treatments. *DS* duplex sequencing; *Min* the minimum mutation counting method; *Max* the maximum mutation counting method

When the data were expressed in terms of the proportion of individual base substitutions, A:T → G:C transition increased in 2D cultures treated with all concentrations of NDMA and in 3D cultures for most concentrations of NDMA, regardless of the sequencing approach and the mutation counting method used (Fig. 5). A significant increase in the proportion of G:C → A:T transition was observed at the highest concentration of 2 mM in 3D spheroids when the minimum mutation counting method was used for DS (Fig. 5B). In addition, the proportions of G:C → C:G, A:T → T:A, and G:C → T:A transversion were visibly reduced in 2D and/or 3D cultures compared to the vehicle control.

### Trinucleotide spectrum

**Fig. 6:**
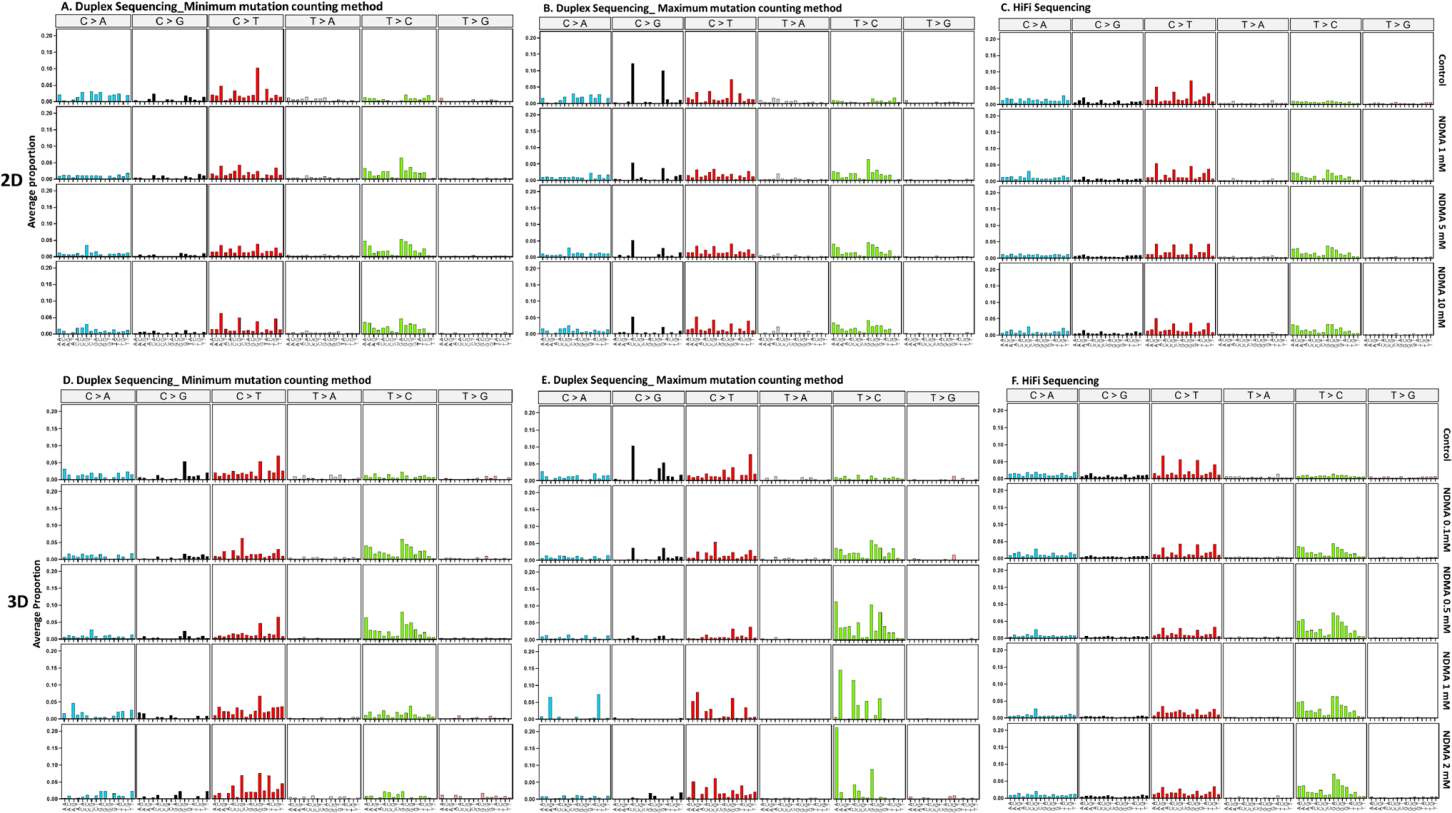
Trinucleotide mutation spectra in NDMA-treated 2D and 3D HepaRG cultures. Data are the average proportion of each base substitution type in all trinucleotide contexts (pyrimidine notation) relative to other base substitution types. **A**–**C**, 2D HepaRG cells; **D**–**F**, 3D HepaRG spheroids

Visual inspection of the trinucleotide spectrum plots showed apparent increases in the proportions of A:T → G:C transitions in NDMA-exposed 2D cultures, regardless of the sequencing approach and the mutation counting method (Fig. 6). In 3D cultures, when the minimum mutation counting method was used in DS, spheroids treated with 0.1 and 0.5 mM NDMA had significant increases in the proportion of A:T → G:C transition, but this increase was not remarkable in spheroids exposed to the two high concentrations (1 and 2 mM) of NDMA. Meanwhile, the proportion of G:C → A:T transition also increased concentration-dependently in the spheroids (Fig. 6D). In contrast, HiFi Sequencing produced apparent increases in the proportion of A:T → G:C transition for all exposed spheroids; similar increases were noted for DS when the maximum mutation counting method was used (Fig. 6E and F).

### Comparison of MF data between DS and HiFi Sequencing

**Fig. 7:**
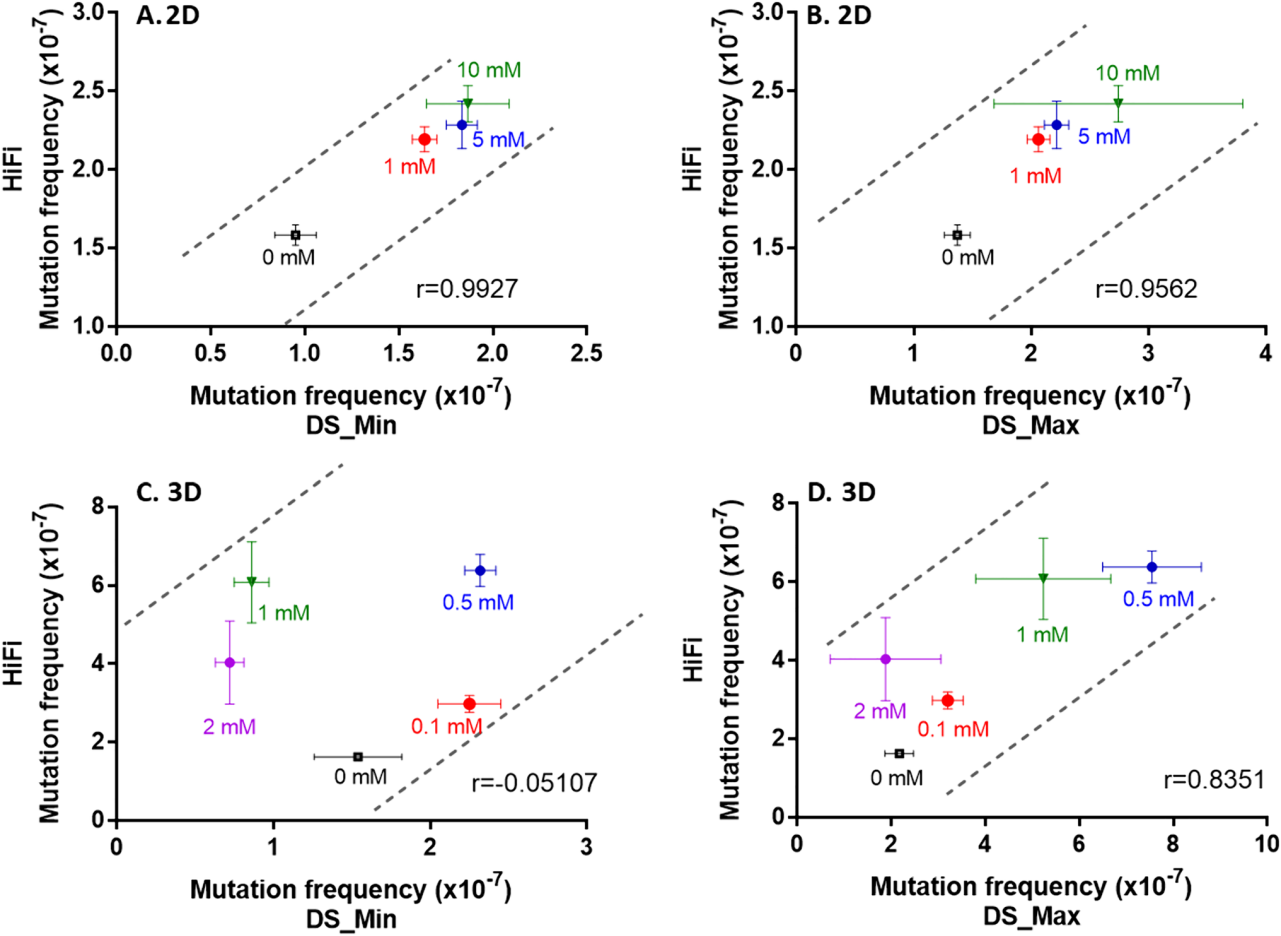
Correlation of mutation frequency obtained from Duplex and HiFi Sequencing in 2D and 3D HepaRG cell cultures. The data are illustrated by the average mutation frequency induced by NDMA and its standard deviation obtained from Duplex Sequencing (DS) against those of HiFi Sequencing. *Min* the minimum mutation counting method; *Max* the maximum mutation counting method

A correlation evaluation of the MF data between DS and HiFi Sequencing was conducted using the Pearson correlation coefficient test. In 2D HepaRG cells, the DS and HiFi Sequencing MF data had a correlation coefficient of > 0.95 (*p* < 0.05) regardless of the mutation counting method in DS, indicating a strong correlation (Fig. 7A and B). In 3D HepaRG spheroids, however, no correlation was observed between MF_DS-Min_ and MF_HiFi_ (Fig. 7C). MF_DS-Max_ and MF_HiFi_ had a correlation coefficient of 0.84, but it was not statistically significant (*p* = 0.08) (Fig. 7D).

## Discussion

Mutation has been proposed as a toxicological endpoint for risk assessment that can serve as a quantitative biomarker of adverse health outcomes (Heflich et al. 2020). To date, the only study that has reported mutation detection in HepaRG cells used whole exome sequencing of cells exposed to a non-cytotoxic concentration of 60 μM retrorsine every 3 days for a total of 45-days (He et al. 2021). The present study investigated a novel strategy for detecting mutations in differentiated, metabolically competent human HepaRG cells using ecNGS. HepaRG cells are bipotent progenitors that are similar to in vivo hepatic progenitors, differentiating into hepatocyte-like and biliary-like cells at confluence (Josse et al. 2008). Due to the absence of p21^CIP1^ and p53 accumulation in differentiated cells, both the hepatocyte-like and biliary-like cells, when seeded at low density, can transdifferentiate into an undifferentiated, elongated cell morphology and can actively divide (Cerec et al. 2007; Guillouzo et al. 2007). Taking advantage of the plasticity of HepaRG cells, we dissociated differentiated HepaRG cells following NDMA treatment and replated the cells at low concentrations in growth medium, which facilitated cell proliferation and the fixation of unrepaired DNA lesions into stable mutations.

In addition to evaluating mutation induction, the present study evaluated the genotoxicity of NDMA following a 72-h treatment in 2D and 3D HepaRG cell models using the comet and MN assays. As was the case with the previous results from 24-h treatments, the 72-h NDMA treatments of 3D cultures produced higher levels of cytotoxicity, DNA strand breaks, and MN formation in the spheroids than in 2D cultures (Fig. 2), likely due to a 2.7-fold increase in *CYP2E1* expression in the spheroids (Seo et al. 2023a, 2023b). It has been noted that species with higher levels of CYP2E1 enzyme have higher sensitivity to NDMA compared to other species (Cross and Ponting 2021). In a comparative DNA damage and repair study in primary hepatocytes derived from 20 human donors and from 20 rats, NDMA induced greater ranges in the amounts of DNA damage and/or unscheduled DNA synthesis than found for other *N*-nitroso compounds (Martelli et al. 1988). The greater effect of NDMA on 3D than 2D HepaRG cells observed in the current study adds to the body of evidence that CYP2E1 plays a significant role in converting NDMA into genotoxic metabolites. Individuals having higher CYP2E1 activities might also be at a higher risk for the mutagenic and/or carcinogenic effects of NDMA exposure.

Detecting mutations in advanced differentiated in vivo-like models is only at a very early stage of development. The present study employed a 72-h treatment with NDMA and observed concentration-dependent increases in MFs in both 2D and 3D HepaRG cell models (Tables 1, 2, 3). The rationale for conducting a 72-h treatment is that NDMA induced DNA damage and MN formation following a 24-h treatment, but an additional incubation was required for detecting the MN in both 2D and 3D models (Guo et al. 2020b; Seo et al. 2023a, 2023b). Previous studies have characterized the proliferative capacity of HepaRG cell models and demonstrated that a portion of cells in HepaRG spheroids express the proliferation marker Ki67 (Clayton et al. 2018) and that bipotent HepaRG hepatocyte-like cells can divide without losing differentiation (Cerec et al. 2007). Differentiated HepaRG cells are DNA repair competent and have a slow proliferation rate compared to undifferentiated HepaRG cells and other mammalian cell models (i.e., HepG2 and TK6 cells), even with hEGF stimulation (van Wenum et al. 2016). It was hypothesized that the 72-h repeat treatment could facilitate the fixation of mutations resulting from unrepaired DNA lesions. Although a short treatment has significant savings with regards to both labor and time, for weaker or unknown mutagens, longer repeated exposure periods (i.e., 7-day, 14-day, or 28-day), with and without a recovery period, may be required for sufficient accumulation of induced mutations to enable their detection.

NDMA is a potent alkylating agent that is metabolized by CYP2E1 to form α-hydroxymethylnitrosamine, which then is converted to the reactive intermediate methyldiazonium ion, an alkylating agent that methylates macromolecules including nucleic acids and proteins (George et al. 2019). The major DNA adducts in liver DNA from rats treated with NDMA are *N*^7^-methylguanine (*N*^7^-MeG), *O*^6^-MeG, and* N*^3^-methyladenine (*N*^3^-MeA), followed by *O*^4^-methylthymine (*O*^4^-MeT) (Beranek 1990; Pegg 1977). *N*^7^-MeG is not significant for point mutation or carcinogenesis due to its spontaneous depurination, while *O*^6^-MeG preferentially pairs with thymine (T) rather than with cytosine (C), resulting in G:C → A:T transitions (Salam et al. 2018; Tan et al. 1994). *N*^3^-MeA and *O*^4^-MeT are also potentially mutagenic, predominantly causing A:T → G:C mutations (Fronza and Gold 2004; Preston et al. 1986). Previous studies showed that G:C → A:T mutation was the major mutation in the esophagus and kidney tumors of NDMA-treated rats (Shiao et al. 1998; Wang et al. 1996). G:C → A:T also was the major mutation in *λlac*Z transgenic mice (Souliotis et al. 1998) and the major rat liver S9-mediated mutation in NDMA-treated *Escherichia coli* (Jiao et al. 1993). However, NDMA induced a 13.4-fold increase in A:T → T:A transversion in the *lacI* gene in the livers of Big Blue transgenic mice, while both control and NDMA-treated mice showed similar percentages of G:C → A:T transitions, with higher proportion of this transition occurring at CpG sites in the control mice compared to the treated mice (Shane et al. 2000).

A:T → G:C transitions, and to a lesser extent G:C → A:T transitions, were identified as the predominant mutations induced by NDMA in both in vitro 2D and 3D HepaRG cultures (Fig. 3). We assume that species differences along with other factors may account for differences between our findings and those reported for the TGR studies. First, *O*^6^-MeG, the adduct responsible for G:C → A:T transitions, is mainly removed by *O*^6^-methylguanine-DNA methyltransferase (MGMT) and MGMT is reported to be 8–10 times more active in human liver fractions than rat liver fractions (Hall et al. 1985). Second, human methyltransferases have a much lower rate for removing *O*^4^-MeT adducts compared to the rat methyltransferases (Zak et al. 1994). Third, *N*^3^-MeA adducts are repaired by alkyladenine glycosylase (AAG)-initiated base excision repair (BER) and AAG levels have remarkable effects on NDMA-induced point mutations and tumors in rodent liver (Fahrer and Christmann 2023; Kay et al. 2021). It has been shown that AAG levels in peripheral blood mononuclear cells vary up to tenfold between individual humans (Fahrer and Christmann 2023). Since the HepaRG cell line is derived from a single donor with unknown AAG levels, this mutation may not be common to all human hepatic cells. Intriguingly, NDMA induced primarily G:C → A:T transitions in human B lymphoblastoid cell lines AHH-1 and MCL-5, both transfected with the human *CYP2E1* gene (Dobo et al. 1998). It is worth noting that the mutational spectrum in this study was derived from phenotypically selected mutants at two reporter genes, *TK* and *HPRT*, whereas our study analyzed a larger portion of the genome using a non-selection method for identifying mutations, and this may produce a broader assessment of NDMA-induced mutations in metabolically competent human cells.

Interestingly, when the default minimum mutation counting method was used for analyzing DS data in the present study, the MF_DS_Min_s induced by NDMA after a 72-h exposure were significantly increased in both 2D and 3D HepaRG cell models that were exposed to NMDA treatments resulting in low levels of toxicity (< 20%), whereas the MF_DS_Min_s resulting from treatments producing greater levels of toxicity (1 and 2 mM in the 3D spheroids) were actually lower than the MF_DS_Min_ of the negative control cultures (Figs. 2, 3, and Tables 1, 2). We hypothesize that the reduced mutation frequency at high concentrations in the 3D spheroids may be due to how DS data are normally analyzed, i.e., by generating a value that reduces the possibility that clonality occurring during the cell expansion following treatment affects the ‘mutation frequency’ that is generated. DS targets twenty 2.4 kb fragments distributed on 20 chromosomes. To ensure a low sequencing error rate and high sensitivity, a relatively high sequencing duplex consensus depth of ~ 20,000 was used in the current study, combined with setting the maximum value for variant allele frequency (VAF) at 1% (i.e., only variant alleles present in fewer than 1% of cells are considered to be unique sequence changes or mutations) (Salk et al. 2019). Accordingly, analyzing DNA from a relatively large cell population would reduce the possibility of measuring clonally expanded mutations. In the present study, significant cytotoxicity was observed in the 3D spheroids exposed to 1 and 2 mM NDMA for 72 h, and the induced cytotoxicity appeared to become more severe after a 6-day extended incubation (Fig. 2F). We also observed a significant increase in population doubling time in cells treated with high concentrations of NDMA compared to those of the untreated control and low/non-toxic concentrations (data not shown). It should be noted that the spheroids were dissociated and replated as attached cultures following the 72-h treatment and fewer viable cells attached and grew in cultures treated with the higher concentrations of NDMA (1 and 2 mM) due to the higher cytotoxicity compared to untreated cultures and those treated with the lower concentrations of NDMA (0, 0.1, and 0.5 mM). This might produce a ‘bottleneck’ that would tend to promote clonality for any mutations induced in the surviving cell population. Additionally, high levels of toxicity might result in a wider range of damage to treated cells. Consequently, the less damaged cells may be more likely to survive and expand, becoming the predominant populations at confluence. Such “growth selection” would presumably not have impacted greatly on the cultures where no or less cytotoxicity was observed (i.e., the 2D cultures and the low concentrations used to treat the 3D cultures).

To test the hypothesis, we reanalyzed both 2D and 3D DS data using the maximum mutation counting method. The minimum and maximum methods generated similar MFs, simple base substitutions, and trinucleotide spectra in data derived from NDMA treatments of 2D HepaRG cells and data derived from treating 3D spheroids with 0.1 and 0.5 mM NDMA, all the treatments that resulted in little or no toxicity (Tables 1, 2), although there seems to be an outlier for one data point at the 10 mM NDMA in 2D DS_Max (Fig. 3B). Unfortunately, there is no obvious technical or other explanation for the outlier. In contrast, the cytotoxic concentrations of 1 and 2 mM NMDA resulted in much higher MF_DS_Max_s and an increased proportion of A:T → G:C transitions compared to data analyzed by the MF_DS_Min_ method. This is presumably because the maximum method includes clonally expanded mutations which have been filtered out by the minimum method. Our results suggest that severely cytotoxic concentrations should be avoided when DS is used; exactly what constitutes severe cytotoxicity is not clear, and is probably dependent upon the method used for measuring cytotoxicity (in our case the ATP assay and observations made of cell growth following treatment). Ideally, mutagenesis should reflect mutation (not mutant) frequency, and while the maximum method may capture all the sequence changes in a population of cells, it is unable to distinguish between clonally expanded mutations and de novo mutagenesis.

In the present study, we compared two ecNGS technologies using the same set of gDNA extracted from 2D and 3D HepaRG cell cultures exposed to NDMA. DS sequences a small portion (48 kb) of human genome and relies on a large population of cells to measure mutations, and thus, as we discovered, MFs and mutational spectrum are influenced by the cytotoxicity and the method used to count mutations. In contrast, HiFi technology sequences 90% of human genome at a lower depth, which makes the mutation analysis less sensitive to the cytotoxicity of the exposures and less likely to detect the effects of clonality. In the present study, HiFi data generated almost identical mutational spectra in 2D and 3D HepaRG cell cultures exposed to NDMA, regardless of the cytotoxicity of the treatment (Fig. 6C and F). The same spectrum also was observed in DS data derived from treatments with non-toxic concentrations of NDMA (treatments of 2D cells and low concentrations of NDMA in 3D spheroids), but not for treatments resulting in elevated levels of toxicity (1 and 2 mM in 3D cultures) (Fig. 6A, B, D, and E). We speculate that these toxic concentrations may not have resulted in a sufficient number of mutations to provide a full picture of the mutational spectrum due to the reduced cell populations caused by cytotoxicity and the relatively small sequencing target used by DS. Other factors could also account for differences in NDMA-induced mutation spectra between the two approaches, i.e., differences in fragmentation of gDNA (enzymatic vs. mechanical shearing), sequencing methods (short reads vs. long reads), alignment tools, and various bioinformatic filters.

In conclusion, we have demonstrated the feasibility of using differentiated HepaRG cells for mutation detection by employing ecNGS. Other genotoxicity endpoints, i.e., MN formation and DNA damage as measured by the comet assay, the γH2AX assay, and TGx-DDI (toxicogenomic-DNA damage inducing) transcriptomic biomarker analysis have been adapted previously for use with these cells (Barranger and Le Hegarat 2022; Buick et al. 2021; Louisse et al. 2022; Seo et al. 2022, 2023a). This advance enables a full range of genotoxicity endpoints to be monitored in the HepaRG 2D cells and 3D spheroid system, making it a potentially powerful human-based platform for genotoxicity testing. We found that NDMA induces concentration-dependent increases in MF in treated cells at non-toxic concentrations, suggesting that mutation may potentially be used as a biomarker for genotoxicity assessment. Both DS and HiFi Sequencing can be used for detecting mutations and characterizing mutational spectrum in HepaRG cells. Although HiFi Sequencing appeared to be more tolerant of the effects of cytotoxicity, caution should be taken when MFs are evaluated at cytotoxic concentrations using either approach. A potential weakness of the present study is that the health, metabolic capacity, and cell growth of 3D HepaRG spheroids are not measured, although the spheroids have been characterized in our previous studies (Seo et al. 2022, 2023a). Further validation studies on the HepaRG system are necessary for establishing the conditions necessary for confidently measuring mutations in terms of the size of the surviving population and its growth kinetics following treatment with a test substance. This proof-of-principle study may lay a foundation for developing mutation assays with other advanced tissue and differentiated cell models, such as the 3D reconstructed human skin models, complex multi-organ spheroids, and transformative microphysiological systems.

## Data Availability

All data that support the findings of this study are available from the corresponding author upon reasonable request.

## References

[CR1] Ali R, Guo X, Lin H (2014). Mutant frequency in comparison to oxidative DNA damage induced by ochratoxin A in L5178Y tk+/- (3.7.2C) mouse lymphoma cells. Drug Chem Toxicol.

[CR2] Allemang A, Mahony C, Lester C, Pfuhler S (2018). Relative potency of fifteen pyrrolizidine alkaloids to induce DNA damage as measured by micronucleus induction in HepaRG human liver cells. Food Chem Toxicol.

[CR3] Barranger A, Le Hegarat L (2022). Towards better prediction of xenobiotic genotoxicity: CometChip technology coupled with a 3D model of HepaRG human liver cells. Arch Toxicol.

[CR4] Beranek DT (1990). Distribution of methyl and ethyl adducts following alkylation with monofunctional alkylating agents. Mutat Res.

[CR5] Buick JK, Williams A, Gagné R (2020). Flow cytometric micronucleus assay and TGx-DDI transcriptomic biomarker analysis of ten genotoxic and non-genotoxic chemicals in human HepaRG™ cells. Genes Environ.

[CR6] Buick JK, Williams A, Meier MJ (2021). A modern genotoxicity testing paradigm: integration of the high-throughput cometchip(r) and the tgx-ddi transcriptomic biomarker in human heparg cell cultures. Front Public Health.

[CR7] Cerec V, Glaise D, Garnier D (2007). Transdifferentiation of hepatocyte-like cells from the human hepatoma HepaRG cell line through bipotent progenitor. Hepatology.

[CR8] Cho E, Swartz CD, Williams A (2023). Error-corrected duplex sequencing enables direct detection and quantification of mutations in human TK6 cells with strong inter-laboratory consistency. Mutat Res Genet Toxicol Environ Mutagen.

[CR9] Clayton NP, Burwell A, Jensen H (2018). Preparation of three-dimensional (3-D) human liver (HepaRG) cultures for histochemical and immunohistochemical staining and light microscopic evaluation. Toxicol Pathol.

[CR10] Cross KP, Ponting DJ (2021). Developing structure-activity relationships for N-nitrosamine activity. Comput Toxicol.

[CR11] Dobo KL, Eastmond DA, Grosovsky AJ (1998). Sequence specific mutations induced by N-nitrosodimethylamine at two marker loci in metabolically competent human lymphoblastoid cells. Carcinogenesis.

[CR12] Dodge AE, LeBlanc DPM, Zhou G (2023). Duplex sequencing provides detailed characterization of mutation frequencies and spectra in the bone marrow of MutaMouse males exposed to procarbazine hydrochloride. Arch Toxicol.

[CR13] Donato MT, Gallego-Ferrer G, Tolosa L (2022). In Vitro models for studying chronic drug-induced liver injury. Int J Mol Sci.

[CR14] Fahrer J, Christmann M (2023). DNA Alkylation damage by nitrosamines and relevant DNA repair pathways. Int J Mol Sci.

[CR15] FDA (2021) Control of nitrosamine impurities in human drugs. US Food and Drug Administration https://www.fda.gov/regulatory-information/search-fda-guidance-documents/control-nitrosamine-impurities-human-drugs (accessed 24 January 2024)

[CR16] Fronza G, Gold B (2004). The biological effects of N3-methyladenine. J Cell Biochem.

[CR17] George J, Tsuchishima M, Tsutsumi M (2019). Molecular mechanisms in the pathogenesis of N-nitrosodimethylamine induced hepatic fibrosis. Cell Death Dis.

[CR18] Guillouzo A, Corlu A, Aninat C, Glaise D, Morel F, Guguen-Guillouzo C (2007). The human hepatoma HepaRG cells: a highly differentiated model for studies of liver metabolism and toxicity of xenobiotics. Chem Biol Interact.

[CR19] Guo X, Seo JE, Li X, Mei N (2020). Genetic toxicity assessment using liver cell models: past, present, and future. J Toxicol Environ Health Part B.

[CR20] Guo X, Seo JE, Petibone D (2020). Performance of HepaRG and HepG2 cells in the high-throughput micronucleus assay for in vitro genotoxicity assessment. J Toxicol Environ Health A.

[CR21] Hall J, Bresil H, Montesano R (1985). O6-Alkylguanine DNA alkyltransferase activity in monkey, human and rat liver. Carcinogenesis.

[CR22] He Y, Shi M, Wu X (2021). Mutational signature analysis reveals widespread contribution of pyrrolizidine alkaloid exposure to human liver cancer. Hepatology.

[CR23] Heflich RH, Johnson GE, Zeller A (2020). Mutation as a toxicological endpoint for regulatory decision-making. Environ Mol Mutagen.

[CR24] IARC (1978). Some N-nitroso compounds. IARC Monogr Eval Carcinog Risks Hum.

[CR25] Jalili P, Huet S, Burel A (2022). Genotoxic impact of aluminum-containing nanomaterials in human intestinal and hepatic cells. Toxicol in Vitro.

[CR26] Jiao J, Glickman BW, Anderson MW, Zielinska M (1993). Mutational specificity of N-nitrosodimethylamine: comparison between in vivo and in vitro assays. Mutat Res.

[CR27] Josse R, Aninat C, Glaise D (2008). Long-term functional stability of human HepaRG hepatocytes and use for chronic toxicity and genotoxicity studies. Drug Metab Disposition.

[CR28] Josse R, Rogue A, Lorge E, Guillouzo A (2012). An adaptation of the human HepaRG cells to the in vitro micronucleus assay. Mutagenesis.

[CR29] Kay JE, Corrigan JJ, Armijo AL (2021). Excision of mutagenic replication-blocking lesions suppresses cancer but promotes cytotoxicity and lethality in nitrosamine-exposed mice. Cell Rep.

[CR30] Kirkland D, Pfuhler S, Tweats D (2007). How to reduce false positive results when undertaking in vitro genotoxicity testing and thus avoid unnecessary follow-up animal tests: report of an ecvam workshop. Mutat Res.

[CR31] Kirkland D, Kasper P, Martus HJ (2016). Updated recommended lists of genotoxic and non-genotoxic chemicals for assessment of the performance of new or improved genotoxicity tests. Mutat Res Genet Toxicol Environ Mutagen.

[CR32] Li X, Chen S, Guo X (2020). Development and application of TK6-derived cells expressing human cytochrome P450s for genotoxicity testing. Toxicol Sci.

[CR33] Louisse J, Mulder PPJ, Gerssen A (2022). Bioassay-directed analysis-based identification of relevant pyrrolizidine alkaloids. Arch Toxicol.

[CR34] Mandon M, Huet S, Dubreil E, Fessard V, Le Hegarat L (2019). Three-dimensional HepaRG spheroids as a liver model to study human genotoxicity in vitro with the single cell gel electrophoresis assay. Sci Rep.

[CR35] Marchetti F, Cardoso R, Chen CL (2023). Error-corrected next-generation sequencing to advance nonclinical genotoxicity and carcinogenicity testing. Nat Rev Drug Discov.

[CR36] Martelli A, Robbiano L, Gazzaniga GM, Brambilla G (1988). Comparative study of DNA damage and repair induced by ten N-nitroso compounds in primary cultures of human and rat hepatocytes. Cancer Res.

[CR37] Mei N, Guo X, Moore MM, Caldwell GW, Yan Z (2014). Methods for using the mouse lymphoma assay to screen for chemical mutagenicity and photo-mutagenicity. Optimization in Drug Discovery.

[CR38] Miranda JA, McKinzie PB, Dobrovolsky VN, Revollo JR (2022). Evaluation of the mutagenic effects of Molnupiravir and N4-hydroxycytidine in bacterial and mammalian cells by HiFi sequencing. Environ Mol Mutagen.

[CR39] Miranda JA, Fenner K, McKinzie PB, Dobrovolsky VN, Revollo JR (2023). Unbiased whole genome detection of ultrarare off-target mutations in genome-edited cell populations by HiFi sequencing. Environ Mol Mutagen.

[CR40] Obach RS, Dobo KL (2008). Comparison of metabolite profiles generated in Aroclor-induced rat liver and human liver subcellular fractions: considerations for in vitro genotoxicity hazard assessment. Environ Mol Mutagen.

[CR41] OECD (2016a) OECD Guidelines for the Testing of Chemicals, Section 4. OECD Publishing, Paris https://www.oecd-ilibrary.org/environment/oecd-guidelines-for-the-testing-of-chemicals-section-4-health-effects_20745788 (accessed 24 January 2024) doi:10.1787/20745788

[CR42] OECD (2016b) Test No. 487: In Vitro Mammalian Cell Micronucleus Test. OECD Guidelines for the Testing of Chemicals, Section 4, OECD Publishing, Paris 10.1787/9789264264861-en: (accessed 24 January 2024) doi: 10.1787/9789264264861-en

[CR43] OECD (2015) Guidance document on revisions to OECD genetic toxicology test guidelines. OECD Workgroup of National Coordinators for Test 42 Guidelines (WNT) https://www.oecd.org/env/ehs/testing/Draft%20Guidance%20Document%20on%20OECD%20Genetic%20Toxicology%20Test%20Guidelines.pdf (accessed 24 January 2024)

[CR44] Paul P, Malakar AK, Chakraborty S (2019). The significance of gene mutations across eight major cancer types. Mutat Res Rev Mutat Res.

[CR45] Pegg AE (1977). Formation and metabolism of alkylated nucleosides: possible role in carcinogenesis by nitroso compounds and alkylating agents. Adv Cancer Res.

[CR46] Preston BD, Singer B, Loeb LA (1986). Mutagenic potential of O4-methylthymine in vivo determined by an enzymatic approach to site-specific mutagenesis. Proc Natl Acad Sci USA.

[CR47] Revollo JR, Miranda JA, Dobrovolsky VN (2021). PacBio sequencing detects genome-wide ultra-low-frequency substitution mutations resulting from exposure to chemical mutagens. Environ Mol Mutagen.

[CR48] Robison TW, Jacobs A (2009). Metabolites in safety testing. Bioanalysis.

[CR49] Salam T, Premila Devi S, Duncan Lyngdoh RH (2018). Molecular criteria for mutagenesis by DNA methylation: Some computational elucidations. Mutat Res.

[CR50] Salk JJ, Kennedy SR (2020). Next-generation genotoxicology: using modern sequencing technologies to assess somatic mutagenesis and cancer risk. Environ Mol Mutagen.

[CR51] Salk JJ, Schmitt MW, Loeb LA (2018). Enhancing the accuracy of next-generation sequencing for detecting rare and subclonal mutations. Nat Rev Genet.

[CR52] Salk JJ, Loubet-Senear K, Maritschnegg E (2019). Ultra-sensitive tp53 sequencing for cancer detection reveals progressive clonal selection in normal tissue over a century of human lifespan. Cell Rep.

[CR53] Seo JE, He X, Muskhelishvili L (2022). Evaluation of an in vitro three-dimensional HepaRG spheroid model for genotoxicity testing using the high-throughput CometChip platform. Altex.

[CR54] Seo JE, Li X, Le Y, Mei N, Zhou T, Guo X (2023). High-throughput micronucleus assay using three-dimensional HepaRG spheroids for in vitro genotoxicity testing. Arch Toxicol.

[CR55] Seo JE, Yu JZ, Xu H (2023). Genotoxicity assessment of eight nitrosamines using 2D and 3D HepaRG cell models. Arch Toxicol.

[CR56] Shane BS, Smith-Dunn DL, de Boer JG, Glickman BW, Cunningham ML (2000). Mutant frequencies and mutation spectra of dimethylnitrosamine (DMN) at the lacI and cII loci in the livers of Big Blue transgenic mice. Mutat Res.

[CR57] Shiao YH, Rice JM, Anderson LM, Diwan BA, Hard GC (1998). von Hippel-Lindau gene mutations in N-nitrosodimethylamine-induced rat renal epithelial tumors. J Natl Cancer Inst.

[CR58] Souliotis VL, van Delft JH, Steenwinkel MJ, Baan RA, Kyrtopoulos SA (1998). DNA adducts, mutant frequencies and mutation spectra in lambda lacZ transgenic mice treated with N-nitrosodimethylamine. Carcinogenesis.

[CR59] Souton E, Severin I, Le Hegarat L (2018). Genotoxic effects of food contact recycled paperboard extracts on two human hepatic cell lines. Food Addit Contam Part A Chem Anal Control Expo Risk Assess.

[CR60] Tan HB, Swann PF, Chance EM (1994). Kinetic analysis of the coding properties of O6-methylguanine in DNA: the crucial role of the conformation of the phosphodiester bond. Biochemistry.

[CR61] Tascher G, Burban A, Camus S (2019). In-depth proteome analysis highlights HepaRG cells as a versatile cell system surrogate for primary human hepatocytes. Cells.

[CR62] van Wenum M, Adam AA, Hakvoort TB (2016). Selecting cells for bioartificial liver devices and the importance of a 3d culture environment: a functional comparison between the heparg and c3a cell lines. Int J Biol Sci.

[CR63] Verheyen GR, Deun KV, Miert SV (2017). Testing the mutagenicity potential of chemicals. J Genet Genome Res.

[CR64] Wang D, Weghorst CM, Calvert RJ, Stoner GD (1996). Mutation in the p53 tumor suppressor gene in rat esophageal papillomas induced by N-nitrosomethylbenzylamine. Carcinogenesis.

[CR65] Wang Y, Mittelstaedt RA, Wynne R (2021). Genetic toxicity testing using human in vitro organotypic airway cultures: Assessing DNA damage with the CometChip and mutagenesis by Duplex Sequencing. Environ Mol Mutagen.

[CR66] Zak P, Kleibl K, Laval F (1994). Repair of O6-methylguanine and O4-methylthymine by the human and rat O6-methylguanine-DNA methyltransferases. J Biol Chem.

